# Adverse Outcomes of Travel-Related Cosmetic Procedures among US Residents, 2014–2024

**DOI:** 10.3201/eid3206.251883

**Published:** 2026-06

**Authors:** Kiara McNamara, Adam Rowh, Rhett Stoney, Kiran M. Perkins

**Affiliations:** Centers for Disease Control and Prevention, Atlanta, Georgia, USA

**Keywords:** healthcare-associated infections, cosmetic procedures, travel, medical tourism, bacteria, United States

## Abstract

We describe infections and other adverse outcomes among US residents who traveled for cosmetic procedures within the United States or abroad during 2014–2024. Outbreaks of adverse events related to such procedures often involve multiple states and geographically separated patients, making outbreak detection and investigation challenging.

Reports of adverse outcomes among persons who travel across domestic and international borders for planned medical care are reported globally (*1*–*4*). Cosmetic procedures remain a prominent category of planned medical care for which patients travel (*1*,*3*,*5*). The number of persons who travel for cosmetic procedures is unknown but is predicted to increase as demand for low-cost procedures rises and services that enable destination-based medical care expand (*3*,*6*). Additional motivators to travel for cosmetic procedures might include shorter wait times, preference for culturally similar providers, perceived quality of care, desired cultural appearances, and convenience of combining procedures with leisure travel. We reviewed Centers for Disease Control and Prevention (CDC) consultations with local and state jurisdictions to describe risks associated with traveling for cosmetic procedures among US residents.

## The Study

CDC’s Division of Healthcare Quality Promotion (DHQP) (National Center for Emerging and Zoonotic Infectious Diseases) supports health departments investigating reports of patient harm resulting from breaches in healthcare infection prevention and control (IPC) through consultation. We defined consultation as a verbal or written request to CDC from health departments for investigation and technical assistance. We reviewed DHQP consultation records for the period of January 1, 2014–December 31, 2024, using specific search terms (Appendix) to identify investigations in which US residents traveled for cosmetic procedures. We included consultations in which US residents traveled (i.e., within or outside the United States) for cosmetic procedures that involved an adverse outcome. We excluded consultations involving US residents who traveled for nonmedical purposes; received incidental, noncosmetic medical care; or underwent reconstructive or bariatric surgery.

We abstracted information regarding the number of patients, country or state where the procedure occurred, type of procedure performed, reporting jurisdiction(s), infection type, pathogen(s), healthcare setting, postsurgical interventions, treatment of complications, clinical outcomes, and findings from IPC assessments where available. Two authors (K.M. and A.R.) reviewed included consultations and 10% of excluded consultations for agreement and jointly abstracted the above variables to ensure consistency.

Of 2,162 total consultations, 34 consultations involved patients who traveled for medical care. Of those, 21 consultations involved ≈145 patients who traveled for cosmetic procedures and were included. US residents traveled to international (n = 17) and domestic destinations (n = 4) and underwent liposuction (n = 12), abdominoplasty (n = 9), and other cosmetic procedures (Table 1). Sixteen consultations included >1 cosmetic procedure. The number of patients per consultation ranged from 1 to 38 patients, although 12 consultations involved only 1 patient. Seven (range 2–20) consultations involved patients from multiple states who underwent cosmetic procedures in international (n = 5) and domestic locations (n = 2) and were associated with the same provider, procedure location, or type of procedure during a specific time (Table 2).

**Table 1:** Characteristics of consultations related to travel by US residents for elective cosmetic medical procedures reported to the Centers for Disease Control and Prevention, 2014–2024

Characteristics	No. consultations, N = 21
Location of procedure	
International	17
United States	4
Setting*	
Surgery center or surgery clinic	14
Med-spa	1
Unknown	6
Type of cosmetic procedure†	
Liposuction with or without fat transfer	12
Abdominoplasty	9
Gluteal augmentation	7
Breast augmentation	6
Other cosmetic surgery‡	4
Unspecified cosmetic medical procedure	4
Pathogen, n = 20§	
Nontuberculous mycobacteria	12
Suspected nontuberculous mycobacteria¶	1
Methicillin-resistant *Staphylococcus aureus*	1
*Fusarium solani* complex	1
Carbapenem-resistant *Pseudomonas aeruginosa*	1
Unknown	4
Type of infection, n = 20§	
Surgical site infection	16
Central nervous system infection	1
Sepsis	1
Unknown site	2
Postsurgical procedural intervention, n = 20#	
Surgical or procedural treatment**	7
Other	5
Antibiotic treatment	7
Outcomes	
Hospitalization	4
Death	4
Unknown	14

*Categorization of setting types was based on descriptions relayed by public health jurisdictions and partners and may have variable interpretations in international and domestic locations. †Types of cosmetic medical procedures were not mutually exclusive. ‡Other types of procedures were upper and lower blepharoplasty, facial fat grafting, lip filler, zygoma reduction-plasty, “thread lift,” dimpleplasty, removal of gluteal implants, panniculectomy, and rhytidectomy. §One consultation was excluded from the denominator of this section because investigation indicated patient outcomes were due to fat emboli and unrelated to an infectious etiology. ¶Suspected nontuberculous mycobacterium were defined as growth on acid-fast bacillus culture without mycobacterial species identification. #Surgical or procedural interventions were not mutually exclusive. **Surgical or procedural treatments include incision and drainage, surgical debridement, and therapeutic surgical revisions.

**Table 2:** Examples of select consultations and adverse outcomes related to travel for elective cosmetic procedures reported to Centers for Disease Control and Prevention, 2014–2024*

Location of procedures, y	Pathogen(s)	No. patients	Procedure(s)	Description	Outcome(s)
Dominican Republic, 2017	NTM	52	Liposuction with and without fat transfer; breast augmentation; abdominoplasty	Multiple strains of NTMs identified, suggesting widespread infection prevention and control lapses across multiple clinics and surgical providers. Extensive case-finding methods used, including call for cases through Epidemic Information Exchange, state-based health alert systems, Infectious Disease Society of America’s Emerging Infections Network, and American Society of Plastic Surgeons’ email distribution list (*14*)	>11 patients received >1 antibiotic; >14 patients required therapeutic surgical procedures; 1 death reported
		
			
Florida, USA, 2017	NTM	3	Gluteal augmentation	3 patients from 2 states developed surgical site infections attributed to NTM after cosmetic procedures	Outcomes not documented
				Procedures completed sequentially on same day by same surgeon in rented operating room	
Dominican Republic, 2018	Methicillin-resistant *Staphylococcus aureus*	3	Abdominoplasty; gluteal augmentation; liposuction	3 patients from same state of residence developed surgical site infections after cosmetic procedures performed at 3 surgical centers abroad by 3 surgeons. 1 clinic associated with prior healthcare-associated outbreak of NTM. Wound cultures from 1 patient identified methicillin-resistant *Staphylococcus aureus. *No other pathogens reported	Outcomes not documented
				
				
Florida, USA, 2022–2023	NTM	19	Gluteal augmentation; liposuction with and without fat transfer; abdominoplasty	Postsurgical infections were identified among 19 patients; of those, 15 patients from 9 states underwent cosmetic procedures at the same clinic and met the investigation’s definition of a confirmed case (*2*). *Mycobacterium abscessus* was identified from wound cultures from all confirmed cases. Case-finding methods included a national call for cases through CDC Epidemic Information Exchange. Surgery center where procedures were performed was closed, and therefore, infection control practices could not be evaluated. Evaluation of infection control practices at another clinic where the same staff worked identified gaps in personal protective equipment use, medical device reprocessing, and environmental cleaning and disinfection	>6 case-patients required intravenous antibiotics; >1 patient hospitalized; >4 case-patients required additional interventions, including computed tomography–guided percutaneous abscess drainage, needle aspiration, incision and drainage, wound debridement, and surgical skin excision with drainage
		
			

*Consultations were defined as a verbal or written request to CDC from health departments for investigation and technical assistance in response to reports of patient harm resulting from breaches in healthcare infection prevention and control. CDC, Centers for Disease Control and Prevention; NTM, nontuberculous mycobacterium.

Postsurgical infections were described in 20 consultations, of which 12 identified suspected or confirmed nontuberculous mycobacteria (NTM). Suspected NTMs were defined as growth on acid-fast bacillus culture without mycobacterial species identification. Surgery centers or surgery clinics (n = 14) were the most frequently reported healthcare setting. Four consultations reported patient deaths (*7*). IPC findings available from 1 domestic and 1 international consultation highlighted gaps in environmental cleaning practices, use of personal protective equipment, hand hygiene, and reprocessing of surgical equipment.

## Conclusions

We identified adverse outcomes among US residents who traveled outside their state of residence for cosmetic procedures within the United States or abroad. This analysis highlights potential for lapses in infection control to contribute to transmission both internationally and domestically and the challenges of detecting and investigating outbreaks in which patients travel. Our findings reinforce the importance of risk mitigation strategies, including effective communication of potential risks to patients, to prevent serious complications of medical procedures or surgeries. CDC provides guidance and information for healthcare personnel and patients who are considering medical tourism abroad (*3*,*8*). US residents might also benefit from those risk mitigation strategies when choosing a domestic destination for cosmetic procedures.

Identifying adverse outcomes among persons who travel for cosmetic procedures is challenging and relies on clinical vigilance and reporting to public health authorities. Approximately one third of consultations reported large outbreak investigations involving multiple patients and states, which often requires extensive case-finding methods and information networks to connect information about geographically dispersed patients (*2*,*7*,*9*). One CDC Data (https://www.cdc.gov/data-modernization/php/one-cdc-data-platform/index.html) is a unified data platform that enables communication among public health professionals. Improved surveillance, continued partnership among clinicians and public health agencies, and strong patient engagement can encourage early identification of adverse outcomes and inform targeted prevention efforts for those settings (*10*,*11*). CDC is working with partners to explore opportunities to improve surveillance of adverse outcomes linked to medical tourism.

More than half of consultations were attributed to NTM, accounting for all domestic consultations and most international consultations. The invasive nature of cosmetic procedures and potential exposure to nonsterile ice and water, which have been associated with NTM outbreaks in healthcare settings, might contribute to the large proportion of travel-related cosmetic consultations involving NTMs (*1*,*10*,*12*). Clinicians might be more likely to report difficult-to-treat and rare infections such as NTM than other infection types. Although NTMs are not nationally notifiable, public health experts recommend investigating even a single case of healthcare-associated extrapulmonary NTM (*13*). Observing IPC practices and evaluating clinical use of nonsterile water or ice before, during, and after cosmetic procedures is a high-yield action for those investigations.

Regulation for facilities where cosmetic procedures are performed can vary, creating challenges to ascertain IPC standards and ensure patient safety. US state regulations can differ by locality, and not all facilities are federally regulated by the Centers for Medicare and Medicaid Services, potentially contributing to varying IPC and water management practices. The existence of international IPC regulations can be unclear, given the complexities of different regulatory frameworks by country. As a result, IPC standards in other countries might be unknown or uncertain. When adverse outcomes related to medical tourism have been identified in the United States, CDC has connected with country public health authorities, such as ministries of health, to alert international partners and help address IPC concerns, decreasing future risk to patients (*7*,*9*). Persistent IPC gaps contribute to a higher risk for infection, and addressing those gaps should be prioritized to ensure safety of cosmetic procedures.

The first limitation of our review is that we used a convenience sample from health departments that requested CDC consultation, and findings do not represent all travel-related healthcare outbreaks. Full investigation details, including specific IPC issues potentially contributing to transmission, were not systematically provided to CDC and, therefore, might be missing or incomplete in CDC records. Outbreaks were likely underdetected and underreported because facilities might not track patient outcomes and reporting standards vary by jurisdiction. Last, we did not include consultations involving cosmetic procedures occurring within a patient’s state of residence, which could pose similar infectious risks.

Outbreaks among persons who travel for cosmetic procedures, regardless of destination, might be difficult to detect and can lead to complex investigations associated with special challenges. Patients should consider the potential risks when traveling for cosmetic procedures. Clinicians should consider risk for infection, particularly NTM, among this population, and have a low threshold for notifying health departments. In addition to notifying the state or local health department, CDC requests notification of complications related to medical tourism by emailing medicaltourism@cdc.gov.

Appendix Additional information about adverse outcomes of travel-related cosmetic procedures among US residents, 2014–2024
