# Supplementary material for: Adverse Outcomes of Travel-Related Cosmetic Procedures among US Residents, 2014–2024
Source: Emerg Infect Dis. 2026 Jun;32(6):1003–7. doi: 10.3201/eid3206.251883 (PMC13245224; doi:10.3201/eid3206.251883)
Supplement: Appendix — Additional information about adverse outcomes of travel-related cosmetic procedures among US residents, 2014–2024 [file 25-1883-Techapp-s1.pdf]

Article DOI: https://doi.org/10.3201/eid3206.251883

EID cannot ensure accessibility for supplementary materials supplied by authors. Readers who have difficulty accessing supplementary content should contact the authors for assistance.

Adverse Outcomes of Travel-Related Cosmetic Procedures among US Residents, 2014–2024

Appendix

Appendix Table. Search terms used to query Division of Healthcare Quality Promotion consultation records to identify investigations of patient travel for cosmetic procedures, CDC, 2014–2024

Travel-terms	Cosmetic terms
Border	Abdominoplasty
Domestic	Botox
International	Brazilian butt lift and “BBL”
Medical tourism	Breast
Resident	Breast augmentation
State lines	Breast reconstruction
Travel	Cosmetic
United States and “U.S.”	Elective
Border	Facial
	Filler
	Liposuction
	Med-Spa
	Plastic surgeon
	Plastic surgery
	Spa
	Tummy tuck
